# Characterizing Psychiatric Disorders Through Graph Neural Networks: A Functional Connectivity Analysis of Depression and Schizophrenia

**DOI:** 10.1155/da/9062022

**Published:** 2025-08-22

**Authors:** Ji-Won Lee, Ye-Eun Kim, Mikhail Votinov, Minghao Xu, Sun-Young Kim, Munseob Lee, Lisa Wagels, Ute Habel, Han-Gue Jo

**Affiliations:** ^1^School of Electronic and Information Engineering, Kunsan National University, Gunsan, Republic of Korea; ^2^Department of Psychiatry, Psychotherapy and Psychosomatics, Medical Faculty RWTH Aachen University, Aachen, Germany; ^3^Institute of Neuroscience and Medicine (INM 10: Brain Structure Function Relationships), Research Centre Jülich, Jülich, Germany; ^4^School of Mechanical Engineering, Kunsan National University, Gunsan, Republic of Korea; ^5^AI Convergence Research Section, Electronics and Telecommunications Research Institute, Gwangju, Republic of Korea; ^6^Department of AI Convergence, College of Computer and Software, Kunsan National University, Gunsan, Republic of Korea

## Abstract

Major depressive disorder (MDD) and schizophrenia (SZ) are among the most debilitating psychiatric disorders, characterized by widespread disruptions in large-scale brain networks. However, the commonalities and distinctions in their large-scale network distributions remain unclear. The present study aimed to leverage advanced deep learning techniques to identify these common and distinct patterns, providing insights into the shared and disorder-specific neural mechanisms underlying MDD and SZ. Recent advances in graph neural networks (GNNs) offer a powerful framework for analyzing brain connectivity patterns, enabling automated learning of complex, high-dimensional network features. In this study, we applied state-of-art GNN architectures to classify MDD and SZ patients from healthy controls (HCs), respectively, using a multisite resting-state fMRI dataset. The attention-based hierarchical pooling GNN (SAGPool) model achieved the highest performance, with mean accuracies of 71.50% for MDD and 75.65% for SZ classification. Using a perturbation-based explainability method, we identified prominent functional connections driving model decisions, revealing distinct patterns of the large-scale network disruption across disorders. In MDD, alterations were dominantly observed in the default mode network (DMN), whereas SZ exhibited prominent alterations in the ventral attention network (VAN). Notably, specific functional connections identified by our model showed significant correlations with clinical symptoms, particularly positive and general symptoms measured by the positive and negative syndrome scale (PANSS) in SZ patients. Our findings demonstrate the utility of GNNs for uncovering complex connectivity patterns in psychiatric disorders and provide novel insights into the distinct neural mechanisms underlying MDD and SZ. These results highlight the potential of graph-based models as tools for both diagnostic classification and biomarker discovery in psychiatric research.

## 1. Introduction

Depression and schizophrenia (SZ) are two of the most severe psychiatric disorders, marked by profound impairments in cognitive, emotional, and behavioral functioning. Despite their distinct diagnostic criteria, these conditions share considerable overlap in clinical symptoms, particularly in the domains of emotional dysregulation and cognitive deficits [1, 2]. Neuroimaging studies have also identified shared aberrant functioning and organization of large-scale brain network between the two disorders [3, 4]. Resting-state functional magnetic resonance imaging (rs-fMRI) has emerged as a critical tool for investigating the brain network organization in these disorders. However, alongside these commonalities, each disorder also possesses unique pathophysiological mechanisms, highlighting the complexity of their distinct disease processes [5, 6].

Large-scale brain networks, including the default mode network (DMN), salience network (SN), and frontoparietal network (FPN), play pivotal roles in cognitive and emotional regulation. These networks are consistently implicated in psychiatric conditions [3, 7]. The DMN, associated with self-referential and introspective processes, often shows aberrant activity in major depressive disorder (MDD) [8–10]. This dysfunction has been linked to ruminative thought patterns, a defining characteristic of depression [7, 11]. However, it should be noted that these findings are not universally consistent, as some studies report no significant differences in DMN activity between MDD patients and healthy controls (HCs) [12, 13]. In SZ, abnormalities have been often observed in both the DMN and SN. Studies have consistently shown abnormal functional connectivity within the DMN in SZ, including both hyperconnectivity and hypoconnectivity depending on the regions and clinical symptomatology [14, 15]. The SN, which plays a critical role in detecting and processing key environmental stimuli, is integral to maintaining cognitive focus [4, 16–18]. Dysfunction in these networks can disrupt salience detection and self-representation, which are core features of SZ.

Although significant progress has been made in understanding these disruptions in MDD and SZ, classical statistical methods face limitations in capturing the nonlinear and dynamic nature of the brain networks. Furthermore, traditional approaches often rely on predefined regions of interest, potentially overlooking critical brain-wide interactions. This highlights the need for advanced computational techniques capable of unraveling the complex topological properties of brain connectivity.

Recent advances in deep learning, particularly in the application of graph neural networks (GNNs), have opened transformative opportunities for characterizing the brain connectivity at the network level [19]. Unlike traditional machine learning models that rely on manually engineered features, GNNs represent the brain as a network of interconnected regions, enabling automated learning of complex, high-dimensional connectivity patterns directly from neuroimaging data. A prominent subset, graph convolutional networks (GCNs; [20]), has emerged as a particularly effective approach, demonstrating robust performance in classifying MDD [10] and SZ [21] from HCs across large, multisite datasets.

The rapid evolution of GNNs has led to the development of advanced techniques, including graph convolution, attention mechanisms, and decision-level ensembles, which have significantly improved the classification accuracy for MDD versus HCs [22–25] and SZ versus HCs [26–28]. These methodological innovations have enhanced the diagnostic potential of GNNs, offering new opportunities for deepening our understanding of the neural mechanisms underlying psychiatric disorders by enabling the identification of key brain features associated with these conditions.

In this study, we employed advanced graph-based models to identify key neural features within the complex brain networks underlying both MDD and SZ. Specifically, we utilized various types of GNN models such as GCN, attention-based model (GATs; [29]), and attention-based hierarchical graph pooling model (SAGPool; [30]) to uncover intricate topological structures and complex connectivity patterns of the brain system. By applying these models to both MDD and SZ datasets, we directly compared model performances and identified shared and distinct patterns of brain network connectivity within a unified framework. The model achieving the highest classification accuracy underwent further analysis using a perturbation-based feature extraction method, enabling the identification of critical features driving classification decisions. This GNN-based methodology allows for direct comparison of functional connectivity alterations between disorders while analyzing the complex connectivity patterns at a system level.

## 2. Materials and Methods

### 2.1. Data Acquisition and Preprocessing

The rs-fMRI data used in this study were obtained from the SRPBS Multidisorder dataset [31], a comprehensive collection of multisite neuroimaging data. This dataset includes 255 patients with MDD, 147 patients with SZ, and 791 HCs. Additionally, different clinical scales, including the Beck depression inventory-II (BDI-II; [32]) and the positive and negative syndrome scale (PANSS; [33]), were measured, providing clinical characterizations of the patients. It should be noted that the BDI-II was predominantly measured for MDD patients, while the PANSS was primarily used for SZ patients, reflecting their different clinical purposes.

Since the SRPBS dataset contains data from multiple sites with varying scanner models and acquisition protocols, not controlling for site effects could introduce non-biological variability and potentially inflate classification performance [34]. Due to limited overlap in acquisition sites between MDD and SZ patients, we separately analyzed matched datasets of MDD versus HC and SZ versus HC to ensure balanced comparisons and minimize potential confounding variables.

The matching procedure was performed based on key demographic variables including age (range criterion of ±2 years), sex, and sites, resulting in two primary comparison datasets: a matched MDD and HC dataset and a matched SZ and HC dataset. During preprocessing, quality control identified artifacts in some subjects, resulting in 271 subjects being excluded, leaving 922 subjects available for further analysis. As a result, 98 MDD patients and 98 matched HCs (MDD-HC dataset), and 115 SZ patients and 115 matched HCs (SZ-HC dataset) were included in this study. Among HCs, 27 individuals served as controls in both datasets. This matching approach for each disorder ensured demographic consistency across comparison groups, enhancing the validity of subsequent analyses.

Functional data were preprocessed using the CONN toolbox [35], including slice-timing correction, realignment, normalization to MNI space, and spatial smoothing (6 mm FWHM). Denoising was performed using the CompCor method, which regresses out noise components from white matter and cerebrospinal fluid (five components each), motion parameters (12 regressors), identified outlier scans, and constant task effects.

### 2.2. Graph Representation

Graph representation of the rs-fMRI data was constructed based on functional connectivity matrices. These matrices were derived using Fisher-transformed Pearson correlation coefficients between BOLD timeseries across 100 cortical regions defined by the Schaefer et al. [36] atlas. The Schaefer parcellation used in our study is a brain template derived from resting-state fMRI data of 1489 participants, integrating local gradient and global similarity approaches, clustering voxels based on the similarity of their functional connectivity, thereby, maximizing within-parcel functional homogeneity, and each parcel is assigned to one of seven canonical large-scale functional networks [36]. These properties align with our aim to investigate the large-scale network distribution in MDD and SZ. Additionally, we selected the Schaefer 100-parcel template because it offers an optimal balance between anatomical precision and functional relevance while maintaining computational efficiency for our GNN approach.

The individual whole-brain functional connectivity matrix was represented as a graph structure *G* = (*V*, *E*, *W*), where *V* and *E* are sets of nodes and edges, respectively, and *W* is the weighted adjacency matrix. Nodes were defined as the 100 atlas-based brain regions, and node features were represented by vectors of nodal functional connectivity, which capture the pattern of connectivity between each region and all other regions in the brain. To define edges between nodes, we constructed a sparse graph for each individual subject using a *k*-nearest neighbors approach, in which each node was connected to its *k* most strongly correlated neighbors based on functional connectivity. This method yields a sparse yet informative representation of the brain network and is based on the assumption that graph structure may vary across individuals, particularly between patients and HCs.

### 2.3. GNNs

Three types of GNN models were employed to classify the dataset (i.e., MDD-HC dataset and SZ-HC dataset). These GCN, GAT, and SAGPool models (see below) are among the well-established and high-performing GNN architectures [19], leveraging convolution mechanism, attention mechanism, and attention-based hierarchical pooling mechanism, respectively. For each dataset, we used an 8:2 ratio split for training and validation purpose. Hyperparameter tuning for each model was conducted empirically based on prediction accuracy evaluated on the validation set. After determining the optimal hyperparameter combination, we compared the classification performances of the three models. The overall performance was evaluated based on the accuracy observed on the validation data. We employed cross-entropy loss function to quantify the loss and the Adam optimizer to update the model parameters. The training configuration was kept consistent across all models, with a learning rate of 5e−5, a mini-batch size of 32, and 500 epochs.

GCN applies convolution operations directly on graphs by aggregating information from each node's neighbors. This operation enables the model to capture local connectivity patterns and relationships within the whole network [20]. The GCN architecture consists of an input layer, graph convolutional hidden layers, global average pooling layers, and a fully connected layer. Each hidden layer is followed by the rectified linear unit (ReLU) activation function. The fully connected output layer is activated by a softmax function to encode output scalars into predictive probabilities for each class. Our specific GCN implementation utilized two graph convolutional hidden layers with 128 and 64 filters, respectively. To reduce the risk of overfitting, dropout with a rate of 0.3 was applied between hidden layers.

GAT enhances graph representation learning by introducing attention mechanisms that assign different levels of importance to edges during the feature aggregation process [29]. By assigning attention coefficients to neighboring nodes, GAT can focus on the most relevant connections, thereby, uncovering meaningful patterns within a complex network. The GAT architecture of our model consists of an input layer, graph attention convolutional hidden layers, a global average pooling layer, and a fully connected output layer. Each hidden layer is followed by the exponential linear unit activation function. Our specific GAT implementation utilized three graph attention convolutional hidden layers. The first and second layers each contained 64 hidden units with 16 attention heads, while the third layer had 64 hidden units with a single attention head. In GAT, dropout is applied at two distinct levels to prevent overfitting, which is a key feature of this architecture. First, a dropout rate of 0.5 is applied within each layer to the attention mechanism. Then, another dropout rate of 0.6 is applied to the hidden node features after each layer. The final output was obtained through a fully connected layer with a softmax activation function, transforming the results into class probabilities.

Inspired by the self-attention graph pooling technique, SAGPool uses self-attention scores to selectively retain important nodes and edges, enabling the model to reduce graph complexity while preserving meaningful structural information [30]. Our SAGPool model builds upon the hierarchical pooling mechanism, which considers both node features and graph topology to achieve effective representation learning. The architecture consists of graph convolutional layers for capturing local neighborhood information, self-attention-based pooling layers for graph reduction, and fully connected layers for classification. The model incorporates a jumping knowledge module for aggregating multilevel graph representations, allowing it to leverage information from different hierarchical levels. ReLU activation is employed in the graph convolutional layers, and dropout with a rate of 0.2 is applied in the fully connected layers to prevent overfitting. The pooling layers retain the top 80% of nodes based on their self-attention scores, ensuring both computational efficiency and preservation of critical network information.

### 2.4. Identifying Functional Connections Contributing to Classification

To investigate the key functional connectivity features driving the model classification, we employed a perturbation-based explainability method on the best-performing model structure, which achieved the highest validation accuracy during training. This approach builds on the principle that perturbing specific input elements causes substantial changes in the model's output, those elements are likely crucial for the model's decision-making process [37]. To systematically evaluate each connection's importance, we applied this method to all 4950 connections among the 100 brain regions (n*⁣*^*∗*^(n−1)/2) from the original functional connectivity matrices. This approach allowed us to assess each connection's influence on the trained model output, regardless of its inclusion in individual subjects' sparse graphs derived from the *k*-nearest neighbors approach. For each connection, we perturbed its value in the full connectivity matrix by replacing it with a value, defined as three times the highest connection strength observed across all subjects. This approach assumes that if modifying a single connection substantially alters the model's output, that connection likely plays an important role in the decision-making process. The degree of influence was quantified by calculating the root mean squared error (RMSE) between the model outputs of the original and perturbed graphs. A higher RMSE indicated greater model sensitivity to the perturbed connection, suggesting its stronger influence on classification. In this way, we identified critical features by systematically perturbing individual connections and measuring their impact on model predictions.

### 2.5. Satistical Analysis

The performance of GNN models was examined using accuracy, sensitivity, and specificity. The accuracy was determined as the percentage of correctly classified individuals among all subjects. The sensitivity and specificity were used to indicate the percentage of correct classifications in MDD or SZ patients and HC, respectively.

Pearson correlation analysis was used to assess the relationship between the identified functional connections and clinical measures (BDI-II for MDD patients, and positive, negative, and general PANSS for SZ patients). When data violated the normality assumption as determined by the Shapiro–Wilk test, Spearman correlation was applied instead. The significance level was set at α = 0.05, and the significance level was adapted for multiple comparisons using Bonferroni correction.

## 3. Results

### 3.1. Classification Performance

**Figure 1:**
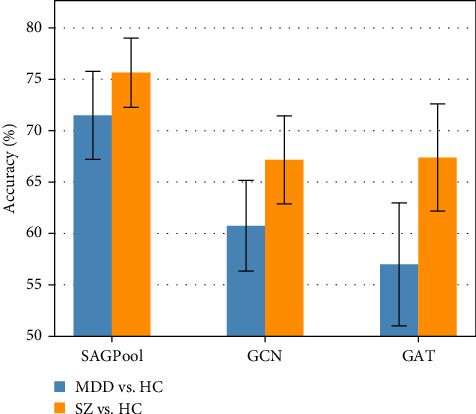
Mean classification accuracy across 10 model runs for different GNN architectures. Performance of SAGPool, GCN, and GAT models is presented for both MDD vs. HC and SZ vs. HC classification tasks. Error bars represent standard deviation across runs.

For MDD versus HC classification, across 10 training runs, the SAGPool model demonstrated superior performance with a mean accuracy of 71.5% ± SD 4.28 (range: 65.0%–77.5%), outperforming both the GCN model with 60.8% ± SD 4.42 (range: 55.0%–70.0%) and the GAT model with 57.0% ± SD 5.99 (range: 47.5%–65.0%). Similarly, in SZ versus HC classification, SAGPool again outperformed with a mean accuracy of 75.7% ± SD 3.37 (range: 69.6%–80.4%), compared to the GCN model's 67.2% ± SD 4.28 (range: 63.0%–73.9%) and the GAT model's 67.4% ± SD 5.23 (range: 58.7%–78.2%) (Figure 1). The SAGPool models achieved on average 0.63 sensitivity and 0.80 specificity for MDD classification, and 0.74 sensitivity and 0.76 specificity for SZ classification.

### 3.2. Brian Regions and Functional Connections Contributing to Classification

**Figure 2:**
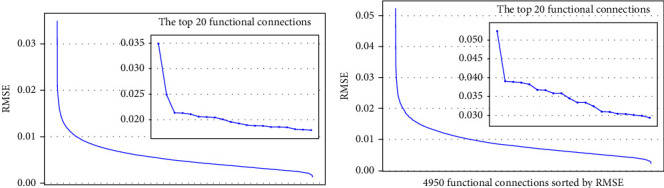
The influence of functional connections on GNN classification. The root mean squared error (RMSE) values represent the degree of influence on the best SAGPool model for (A) MDD and (B) SZ classification. The 4950 functional connections are ranked by their RMSE values. Inset panels highlight the top 20 functional connections.

The best-performing SAGPool models were used to identify the most prominent functional connections that distinguish MDD and SZ patients from HCs, respectively. For both classifications, the top 20 functional connections with higher RMSE values averaged across the 10 trained models (Figure 2) that had greater influence on the models' decision-making process were selected and are presented in Table 1.

**Figure 3:**
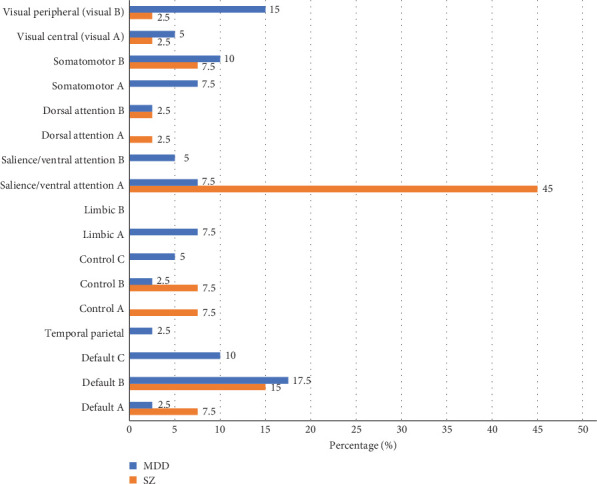
Percentage contribution of network labels in MDD and SZ. Network contributions across brain regions were analyzed using the Schaefer 100-parcel atlas [36], which incorporates the 17-network parcellation scheme [38].

**Figure 4:**
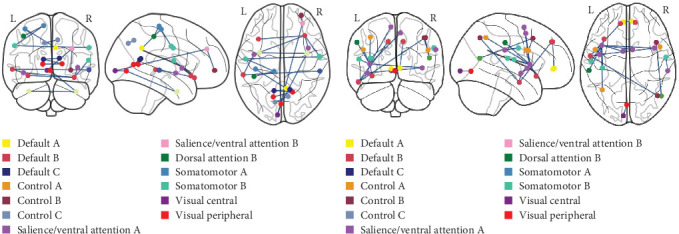
The top 20 prominent functional connections contributing to GNN classification. (A) Functional connections for MDD vs. HC classification, identified by the SAGPool model. (B) Functional connections for SZ vs. HC classification, identified by the SAGPool model. Each node is assigned to one of the 17-networks parcellation scheme [38].

For MDD classification, the most prominent functional connections contributing to the highest-accuracy model (SAGPool) were primarily located in the DMN, mainly including the ventral prefrontal cortex (PFCv) and retrosplenial cortex (Rsp) (Table 1, Figures 3 and 4). Of the 40 brain regions involved in the top 20 significant connections, 12 regions (30%) were from the DMN, 8 regions (20%) from the Visual Network (VN), 7 regions (17.5%) from the Somatomotor Network (SM), 5 regions (12.5%) from the Salience/Ventral Attention Network (VAN), 3 regions each (7.5%) from the Limbic System (LMB) and Control Network (CN), and 1 region each (2.5%) from the Dorsal Attention Network (DAN) and Temporal Parietal Network (TPN).

For SZ classification, the most critical functional connections contributing to the highest-accuracy model (SAGPool) were predominantly located in the VAN, including the insula (Ins), frontal medial cortex (FrMed), and parietal operculum (ParOper) (Table 1, Figures 3 and 4). Among the brain regions involved in the top 20 significant connections, 18 regions (45%) were from the VAN, 9 regions (22.5%) from the DMN, 6 regions (15%) from the CN, 3 regions (7.5%) from the SM, and 2 regions each (5%) from VN and DAN.

### 3.3. Correlation Between Prominent Functional Connections and Clinical Measures

From the study cohort, BDI-II scores were available for 76 of 98 MDD patients, and PANSS scores were available for 104 of 115 SZ patients. These clinical measures were analyzed for correlations with the 20 prominent functional connections identified by the SAGPool models. For MDD classification, no significant correlations between the identified functional connections and BDI-II scores survived Bonferroni correction.

**Figure 5:**
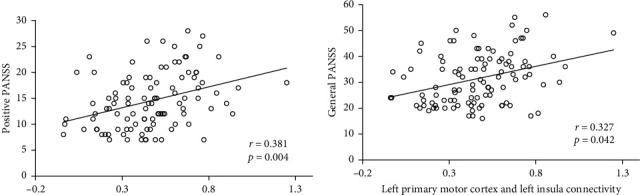
Correlation between notable functional connectivity with positive PANSS (A) and general PANSS (B) scores in individuals with SZ. *p*-Values are Bonferroni corrected.

For SZ patients, the identified functional connection between the left primary sensory cortex of the SM and the left insula of the VAN (specifically between LH_SomMotB_S2_2 and LH_SalVentAttnA_Ins_2) revealed significant correlations with both PANSS positive symptoms (*r* = 0.381, *p*=0.004, Bonferroni-corrected for 3 × 20 comparisons, Figure 5A) and PANSS general psychopathology scores (*r* = 0.327, *p*=0.042, Bonferroni-corrected) (Figure 5B).

## 4. Discussion

This study examined functional connectivity patterns using GNN models and directly compared their performance in characterizing MDD and SZ from HCs, respectively. The results demonstrated the utility of graph-based deep learning models in classifying these psychiatric disorders and identifying key functional connectivity features that contribute to their neural underpinnings. Our findings of common and distinct brain network patterns reinforce the relevance of large-scale brain network disruptions in MDD and SZ, while also advancing methodological approaches for understanding these disorders.

Previous studies have demonstrated that graph-based deep learning methods can effectively identify reliable connectome patterns and accurately classify both MDD [10, 22–25] and SZ [21, 26–28] from HCs. For instance, Liu and Gui [24] achieved an accuracy of 77.5% in MDD classification by integrating brain graph features with phenotypic information across multiple sites, while a recent study on SZ attained an accuracy of 85.8% using a GCN model on a multisite dataset. The classification performance achieved in these studies and our current work with GNN models consistently surpasses that of previous approaches using traditional machine learning methods. These results collectively demonstrate that applying graph-based deep learning approaches to brain connectivity measures represents a promising direction for developing objective diagnostic tools for MDD and SZ.

The superior performance of the SAGPool model architecture compared to GCN and GAT models highlights the importance of hierarchical network representation in analyzing psychiatric disorders. By selectively retaining informative nodes through self-attention mechanisms, SAGPool might have effectively captured the complex patterns of network dysfunction while reducing network complexity. This advantage was particularly evident in the higher sensitivity and specificity achieved for both disorders, suggesting potential clinical utility in diagnostic applications.

The perturbation-based approach employed in this study provided a method for identifying critical functional connections driving GNN model classifications. This methodological approach provides a direct measure of each connection's contribution to the model's classification decisions, rather than relying on correlation-based or post-hoc interpretations. The effectiveness of this approach was demonstrated by its ability to identify biologically plausible and clinically relevant network patterns [19]. In MDD, the method highlighted the predominance of DMN connections, consistent with previous findings linking DMN dysfunction to depressive symptoms [3, 7]. The high proportion of DMN regions (30%) among the significant functional connections supports theories emphasizing the role of aberrant self-referential processing in depression [3, 9].

In SZ, the identification of prominent connections involving the VAN, particularly those responsible for salience detection, sensory processing, and integration, aligns with current understanding of significant processing abnormalities in SZ [4, 16, 18]. The VAN, together with the affective network, is also conceptualized as part of the SN [39]. Dysfunction within this network likely disrupts the normal processes of salience attribution, leading to the misattribution of importance to irrelevant stimuli, a core feature of psychosis [17, 18]. This aligns with the current findings, where connectivity between the insula of VAN and the primary motor cortex of SM showed a significant positive correlation with the severity of positive and general symptoms, as measured by the PANSS (Figure 5). Such a correlation provides a link between aberrant functional connectivity and the clinical manifestations of psychosis, further supporting the hypothesis that SN/VAN disruption underpins the generation of positive symptoms, such as hallucinations and delusions.

The observed alterations in VAN connectivity with SM, particularly with sensory and motor regions, suggest that this dysfunction extends beyond salience detection to also impact sensory integration and the coordination of motor responses. This broader disruption may explain the range of symptoms seen in SZ, including not only psychotic features but also deficits in sensory processing, motor control, and cognitive functioning [18]. Together, these findings emphasize the central role of SN abnormalities in the pathophysiology of SZ and highlight their potential as targets for clinical intervention and biomarker development.

While this study provides valuable insights into the neural mechanisms underlying MDD and SZ through the use of GNNs, several limitations should be acknowledged. First, the datasets used in this study were derived from multisite resting-state fMRI data, which introduces variability due to differences in imaging protocols, scanner hardware, and participant demographics. Although mathcing the age, sex, and measurement sites, residual effects cannot be entirely excluded. Second, although our study includes a relatively large number of participants overall, the sample sizes for the MDD and SZ groups remain modest, particularly when examining subgroups, such as first-episode versus recurrent cases. Additionally, the demographic and clinical characteristics of these groups may not fully capture the broad heterogeneity inherent in MDD and SZ, raising the possibility that our findings may be sample-specific. To address this, validation analyses were conducted using independent public and private datasets. For MDD, a public dataset from Bezmaternykh et al. [40] and a private dataset from Loeffler et al. [41] were employed, comprising resting-state fMRI data from 76 MDD patients and 52 HCs. Applying the same SAGPool model and analytical pipeline as in the SRPBS dataset, these analyses confirmed the consistency of the MDD results, particularly the dominant role of the DMN (40%) in classification. However, the SZ results (122 SZ patients and 119 HCs from the open datasets [42] and [43]) demonstrated a divergent pattern, with key contributions arising from sensory processing networks such as the SM and VN, rather than the VAN that was most prominent in the original SRPBS-based analysis. This discrepancy may reflect sample-specific or population-level differences as well as a stronger heterogeneity in the SZ spectrum disorders and underscores the importance of external validation and cross-population studies, particularly for heterogeneous conditions like SZ. Third, a notable limitation of the present study is that functional network structure is highly dependent on the choice of brain parcellation scheme, which may limit the generalizability of the present findings across different parcellation approaches. To address this, the same analysis was repeated using the Gordon brain template [44], which consists of 333 cortical parcels derived from data-driven clustering of resting-state fMRI signals on the cortical surface. While this additional analysis revealed a consistent dominant contribution of the DMN in MDD classification—aligning with the main results of the present study—it also showed substantial differences in network contributions for SZ classification. Specifically, the VAN, which was prominent in the present original analysis using the Schaefer template, was not similarly represented in the Gordon-based results. This discrepancy likely arises from the fact that, although the DMN is relatively consistently defined across parcellations, the VAN is one of the least consistently labeled and delineated networks, with minimal spatial overlap between the Schaefer and Gordon templates [45]. These findings underscore the importance of careful parcellation selection and multitemplate validation when interpreting functional connectivity results, particularly in heterogeneous disorders such as SZ. Lastly, although perturbation-based explainability methods provided insights into notable functional connections, these methods may not fully capture the complexity of GNN decision-making processes. More comprehensive interpretability frameworks are needed to validate the biological plausibility of the identified features.

## 5. Conclusions

This study demonstrates the potential of GNNs to uncover disorder-specific patterns of brain network disruption in MDD and SZ. By applying a unified GNN-based framework across both diagnostic groups, we directly compared model performance and identified distinct functional connectivity profiles associated with each condition. The DMN emerged as the primary contributor in MDD, aligning with its established role in self-referential thought and affective dysregulation, whereas SZ was characterized by alterations in the VAN, suggesting dysfunction in salience processing and attentional control. These findings support the notion that MDD and SZ are underpinned by differential large-scale network disruptions, with MDD reflecting impairments in introspective processing and SZ associated with aberrant attribution of salience to irrelevant stimuli—a hallmark of psychotic symptoms. Our results underscore the utility of GNNs in advancing data-driven, neurobiologically informed biomarkers for psychiatric classification.

## Figures and Tables

**Table 1:** The top 20 functional connections contributing to GNN classification.

Rank	SAGPool model for MDD classification	SAGPool model for SZ classification
1	LH_VisCent_Striate_1–RH_VisPeri_StriCal_1	LH_SalVentAttnA_Ins_2–RH_SalVentAttnA_Ins_1
2	LH_SalVentAttnA_Ins_1–RH_DefaultB_PFCv_1	LH_SalVentAttnA_Ins_2–RH_SalVentAttnA_FrMed_1
3	LH_DefaultC_Rsp_1–RH_DefaultC_Rsp_1	LH_VisCent_Striate_1–RH_VisPeri_StriCal_1
4	LH_LimbicA_TempPole_1–RH_LimbicA_TempPole_1	LH_SomMotB_S2_2–LH_SalVentAttnA_Ins_2
5	RH_SalVentAttnA_Ins_1–RH_DefaultB_PFCv_1	LH_SalVentAttnA_Ins_1–LH_SalVentAttnA_Ins_2
6	LH_DefaultC_Rsp_1–RH_VisPeri_ExStrInf_1	LH_SalVentAttnA_Ins_1–RH_SalVentAttnA_Ins_1
7	LH_DefaultB_PFCv_1–RH_DefaultB_PFCv_1	LH_SalVentAttnA_Ins_2–LH_SalVentAttnA_FrMed_1
8	RH_VisPeri_StriCal_1–RH_LimbicA_TempPole_1	LH_SalVentAttnA_Ins_2–LH_ContA_IPS_1
9	LH_DefaultB_Temp_2–RH_TempPar_2	LH_DefaultA_PFCm_1–RH_DefaultA_PFCm_1
10	RH_SalVentAttnB_PFCl_1–RH_ContB_PFClv_1	LH_DorsAttnB_PostC_1–LH_SalVentAttnA_Ins_2
11	LH_VisCent_Striate_1–RH_SalVentAttnB_PFCl_1	RH_SomMotB_Cent_1–RH_SalVentAttnA_ParOper_1
12	LH_VisPeri_StriCal_1–LH_DefaultC_Rsp_1	LH_SalVentAttnA_Ins_2–LH_DefaultB_PFCv_1
13	LH_SalVentAttnA_Ins_1–LH_DefaultB_Temp_2	LH_SalVentAttnA_Ins_1–LH_DefaultB_Temp_1
14	RH_SomMotB_S2_2–RH_DefaultB_PFCv_1	LH_SomMotB_S2_1–LH_SalVentAttnA_Ins_2
15	LH_ContC_pCun_1–RH_ContC_pCun_1	LH_DefaultB_Temp_1–LH_DefaultB_PFCd_1
16	LH_SomMotA_2–LH_DorsAttnB_PostC_2	RH_DefaultA_PFCm_1–RH_DefaultB_PFCd_1
17	LH_SomMotB_Cent_1–RH_SomMotB_Cent_1	LH_DefaultB_Temp_1–RH_ContB_IPL_1
18	LH_SomMotA_1–RH_DefaultA_pCunPCC_1	RH_DorsAttnA_ParOcc_1–RH_ContB_PFCld_1
19	LH_SomMotA_2–LH_SomMotB_Cent_1	RH_ContA_PFCl_2–RH_ContB_PFCld_1
20	LH_VisPeri_StriCal_1–RH_VisPeri_ExStrInf_1	LH_SalVentAttnA_Ins_2–LH_ContA_PFCl_2

*Note:* Regions: PFCm, medial prefrontal cortex; PFCd, dorsal prefrontal cortex; PFCld, lateral dorsal prefrontal cortex; PFCl, lateral prefrontal cortex; PFClv, lateral ventral prefrontal cortex; FrMed, frontal medial cortex; pCun, precuneus; pCunPCC, posterior cuneus and posterior cingulate cortex; Rsp, retrosplenial cortex; Ins, insula; Temp, temporal cortex; TempPole, temporal pole; IPL, inferior parietal lobule; ParOper, parietal operculum; ParOcc, parieto-occipital cortex; Cent, central sulcus; PostC, postcentral gyrus; S2, secondary somatosensory cortex; Striate, striate cortex; ExStrInf, inferior extrastriate cortex; StriCal, striate and calcarine cortex; IPS, intraparietal sulcus. The region names are based on the Schaefer 100 atlas [36] with large-scale networks defined by the 17-network parcellation [38].

Abbreviations: LH, left hemisphere; RH, right hemisphere.

## Data Availability

The data that support the findings of this study are available from the corresponding author upon reasonable request.
